# Females Choose Mates Based on Genetic Relatedness in a Small Dasyurid Marsupial, the Agile Antechinus (*Antechinus agilis*)

**DOI:** 10.1371/journal.pone.0122381

**Published:** 2015-04-29

**Authors:** Marissa L. Parrott, Simon J. Ward, Peter D. Temple-Smith, Lynne Selwood

**Affiliations:** 1 Department of Zoology, University of Melbourne, Parkville, Australia; 2 Biodiversity Conservation Division, NT Department of Natural Resources, Environment, the Arts and Sport, Palmerston, Australia; 3 Department of Obstetrics and Gynaecology, Southern Clinical School, Monash University, Clayton, Australia; 4 Wildlife Conservation and Science, Zoos Victoria, Parkville, Australia; University of Padova, ITALY

## Abstract

Females in a variety of taxa mate with more than one male during a single oestrus and exhibit mate preferences for genetically compatible males, but the influence of female mate choice on siring success is not clearly understood. Whether females choose to mate with more than one male or endure forced copulations is also often unknown. Here, we examined the effects of genetic relatedness on female mate choice and siring success in a small semelparous carnivorous marsupial, the agile antechinus (*Antechinus agilis*), during two consecutive breeding seasons. Experimental trials were conducted in captivity over periods of 72 hours using interconnected enclosures in which female antechinus could choose to access any of four separated males, but males were only able to access females that entered their quarters. Females had access to two genetically similar and two genetically dissimilar males simultaneously and all behavioural interactions were observed and scored from continuous video recordings. Genetic similarity between mates and paternity of young was determined by microsatellite analyses. Some females chose to enter and mate with more than one male during a single oestrus period. Although females investigated all males, they spent significantly more time visiting, and mated more times with, genetically dissimilar males. Males that were genetically dissimilar to the female sired 88% of subsequent offspring. Whilst males mated readily with most females, they rejected the advances of some receptive females, indicating a previously unexpected level of male mate choice. The results show that genetic relatedness between mates has a significant influence on mate choice, breeding and siring success in the agile antechinus.

## Introduction

A female’s choice of mate can significantly affect her reproductive success [1]. In social systems that involve no paternal investment other than spermatozoa, females are expected to choose males that confer greater survival and future reproductive success to their offspring (reviewed in [1,2]). Females that are permitted to choose mates in captivity may produce greater quality offspring with improved survival, social dominance, larger home ranges, better nest sites and nests [3] and increased attractiveness as mates [4]. Similarly, in the wild, a female’s choice of mate can lead to increased fitness and parasite resistance in offspring [5]. Females in a variety of taxa may choose males based on a number of criteria, including ‘good’ or compatible genes with a females own genotype, genes of the major histocompatibility complex (MHC) that can offer a reliable olfactory indicator of male health, genetic diversity and quality ([2]), viability genes or genetic relatedness [6,7,8]. While viability genes are often expressed through secondary sexual characteristics, it is less clear how females assess the genetic relatedness or incompatibility of potential mates and how this affects the siring success of individual males [6,1,9,10]. Such information is lacking for numerous species and the mechanisms for multiple mate selection and the effects of female mate preferences on siring success are still poorly understood.

Females mate with more than one male during a single oestrus in a range of species (e.g. common shrews, *Sorex araneus* [11]; Gunnison’s prairie dogs, *Cynomys gunnisoni*, [12]; agile antechinus, *Antechinus agilis*, [13,14]; feathertail gliders, *Acrobates pygmaeus*, [15], saltmarsh sparrows (*Ammodramus caudacutus*; [16]), grass snakes (*Natrix natrix*, [17]), eastern water skinks (*Eulamprus quoyii*; [18]), but it is often difficult to determine whether females choose to mate with more than one male or endure forced copulations. Females that mate with a number of different males potentially face greater risk of injury or disease [19,20], but may benefit through increased reproductive output by ensuring adequate levels of sperm for fertilisation [21,22,18] and/or safeguarding against the possible incompatibility or sterility of some males [2,23]. Females may also rely on competition between spermatozoa from two or more males to fertilise ova and produce the highest quality young [24,25]. Species with multiple mating strategies often produce litters that are sired by more than one male which may increase the success and survival of litters by increasing genetic variability [26] and heterozygosity [6,21].

This research investigated the effects of genetic relatedness between mates on female choice and the outcomes of multiple mating in the agile antechinus. This species is promiscuous [11,27,28] with multiple paternity occurring in 96%–98% of litters and an average of three to four sires per litter ([14], MLP unpub. data). Most males sire young in wild populations with 81% siring offspring in a year where the population was at parity and 100% siring offspring when the population was female biased (MLP unpub. data). Little is known about mate selection in antechinus, but the level of information available on other aspects of their reproduction makes them an ideal model species in which to examine the effects of female preference on multiple matings and siring success. Larger males sire a higher proportion of young in wild populations ([29], MLP unpub. data), but captive studies have shown that females choose mates on other criteria, including scent and genetic relatedness, rather than on male size [30,31]. In wild situations, larger males may secure forced copulations, have increased stamina or travel greater distances to pursue females, or exclude smaller males from mating, and override any opportunity for female mate choice [30]. Sperm precedence, where the male that mates closest to ovulation during oestrous receptivity in females sires the highest proportion of young, also significantly influences paternity success [26,32].

In this study, a series of captive mating trials was conducted in which receptive females were provided with a simultaneous choice of four males, but these males could not follow a female out of his enclosure and could not interact directly with other males. The combination of males within each trial was selected to provide each female with a range of potential mates that were of similar size, but varied in their degree of relatedness to her. This allowed us to analyse female and male mate choice behaviours and interactions, and test the following hypotheses: 1) that females prefer males that are genetically dissimilar to themselves; 2) that female agile antechinus choose to mate with more than one male; and 3) that genetically dissimilar males have a greater siring success than males that are more genetically similar to the female.

## Materials and Methods

### Ethics Statement

This research adhered to Animal Behaviour Society Guidelines for the use of animals and was carried out with ethics approval from the Animal Ethics Sub-Committee at the University of Melbourne (AEC 02181) and under Department of Sustainability and Environment Wildlife permits (10002396 and 10002889).

### Animal maintenance

Agile antechinus were trapped in the Mt Disappointment State Forest, Victoria, in July 2003 (n = 28, 12 males and 16 females) and 2004 (n = 24, 12 males and 12 females) and maintained in captivity as described in Parrott et al. [30,31]. Due to extreme drought conditions during the study, animals were in poor condition (based on comparisons of weight with non-drought years, emaciated appearance and dull, rough fur) when collected [33], but all females used in this study survived and were successfully maintained in captivity. On completion of the mate selection experiments, males were released to their original points of capture, except for any that had reached their natural die-off period. Females remained in captivity until young were born and all were then released in their natal nest-boxes back to the wild at their original points of capture.

### Female choice equipment

**Fig 1:**
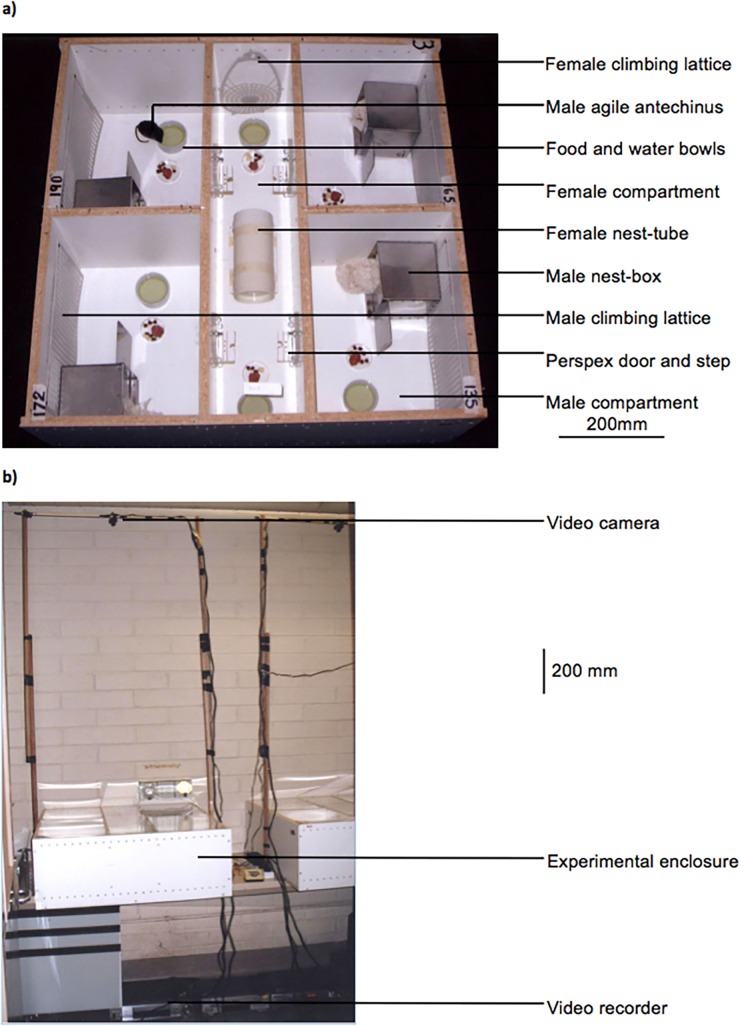
Enclosures for female choice experiments. **(a)** Enclosure seen from above, showing the four male and one female compartments and furnishings. Four outer compartments, with external measurements 400 mm × 300 mm × 300 mm high, each housed a single male and the middle compartment, measuring 800 mm × 200 mm × 300 mm, housed two females. Each male compartment contained a stainless steel nest-box (130 mm × 130 mm × 130 mm) filled with cotton bedding, a cardboard tube, water bowl, feed tray and plastic climbing lattice on one wall. The female compartment contained a nest-tube with cotton bedding (200 mm long × 100 mm diameter) which had entrance/exit holes at each end, plus a water bowl, feed tray and lattice placed at each end. Holes (3 mm diameter) were drilled every 30 mm around the base and top of the four outer walls of the enclosures to allow air flow and in two lines near the base of the walls between the male and female compartments to facilitate movement of animal scents. In the centre of the wall separating each male compartment from the female compartment, a 70 mm × 70 mm gap was covered by a removable clear perspex ‘door’ which contained a 15 mm diameter hole. The size of the hole allowed the exclusion of the larger males which were unable to leave their own compartment in this sexually dimorphic species and allowed almost all females to move in and out of the male and female compartments uninhibited. Females were able to see and interact with males through the perspex and hole. Doors were recessed into a groove across the centre of a wooden ‘door step’ (60 mm × 70 mm × 20 mm high) with grooves on either side of the door to provide grip. **(b)** Video surveillance set-up showing the enclosure, video camera and video recorder.

Experimental enclosures constructed from 16 mm thick white melamine coated particle board (whiteboard panels, Laminex Industries, Tullamarine, Victoria, Australia; n = 3; Fig 1A) were designed with five compartments, one inner containing 2 females and 4 outer each housing a male, which were covered by clear perspex sheets to facilitate observation and video recording. Pairs of females were used as females better adjust to captivity when housed socially (F Kraaijeveld-Smit pers comm). Food was provided in each compartment daily and water (supplemented with Pentavite) was available *ad libitum* [30,31]. All compartments were lined with white paper.

A small black and white closed-circuit digital camera (1/4 B/W G type security surveillance camera, Jaycar, Silverwater, NSW, Australia) suspended above the centre of each enclosure was connected to a video recorder (V-W58H 6 head HiFi VCR, Toshiba, Mt. Waverley, Victoria, Australia; Fig 1B). Light cycles mimicked natural conditions with a dim red light (12 W dark room infrared globe, Philips, North Ryde, NSW, Australia) on during night hours to allow video recording and direct observation. An observer (MLP) was present in the room during all night hours, and most hours during the day, to record direct observations and ensure no animals became trapped or injured. Behaviours were observed via video output on a TV screen or from a distance to minimise disturbance to the animals and ensure animal movements were not influenced. Any females that were seized and held through doors by males and appeared unable to free themselves after ~2 minutes were freed by the observer by gently prodding the male with a light, blunt instrument. This occurred only once when an observer was not present and the female freed herself after ~8 minutes. No females were injured or lost fur when seized. Ambient temperature was maintained at 21 ± 1°C, but temperature was approximately 2°C higher inside the enclosures. Between trials, enclosures were cleaned with detergent, water and 70% ethanol and allowed to air-dry to remove scents and other contaminating material that may have influenced behavioural interactions in the next trial.

### Female choice experiment

In 2003, eight trials using a total of 12 males and 16 females were performed, while in 2004, this was reduced to six trials using 12 males and 12 females. To determine the onset of mating receptivity and ovulation, urine from each female was examined daily to monitor numbers of cornified epithelial cells with ‘Day 0’ of the receptive period corresponding to the time of detection of the first high levels of cornified epithelial cells [34]. Females have a receptive period during which they mate, when numbers of cornified epithelial cell in their urine are high for up to 20 days before ovulation, and continuing after ovulation when such cell numbers start to decline [35]. However, the most fertile receptive period when the percentage of normal embryos is high (60–100%) occurs 5–13 days before ovulation [13] due to declining fertilizing capacity of stored sperm outside that period.

All trials were conducted after day 3 of the receptive period and during the most fertile portion of the receptive period wherever possible (22/28 females; with 3 females paired on days 4–6 and 3 females paired after day 14 due to time constraints), and all were completed prior to ovulation. Male urine was analysed prior to experiments to ensure all males were producing sperm. Females were provided with two males that were more genetically similar and two less genetically similar (dissimilar) to themselves (see below). Females in each pair were identified by black permanent marker on their tails with two thin stripes given to one female and two thick bands given to the other. To remove any influence of male size on mate selection or male success and enable a more controlled examination of female preference for genetic relatedness, males in each trial were selected to be roughly of equal weight, with less than 3 g difference between them (mean ± SE, 2003: 31.8 ± 0.3 g; 2004: 37.7 ± 0.8 g). No males were able to leave their compartments through size exclusion doors. Females chosen for this experiment were in their first breeding season and had not previously mated (mean weight ± SE, 2003: 20.1 ± 0.4 g; 2004: 18.9 ± 0.6 g). Females that attempted to enter areas and were observed to insert a head and torso, but could not enter due to the width of their pelvis (n = 3), were placed with males and observed at all times. This occurred only once while an observer was not present one afternoon, but the female was introduced to the male compartment when she tried to enter again that night. When females attempted to leave, they were removed from the male compartment by the experimenter (MLP), who was present at all times the female was in the compartment. There was no difference in the mating behaviour or breeding success rates of these females compared with females that could enter and leave of their own accord (n = 25). Primiparous females were chosen for this experiment as few females survive to produce a litter in a second year, with no second-year females producing a litter during drought [33]. Each trial was conducted over 72 hours (three days) with constant video recording, providing around 1008 hours of video for analysis. Males were allowed one day rest between trials.

Videos were analysed to determine for each female 1) the number of visits to each male door; 2) the time spent investigating each male; 3) which male compartments she entered; 4) the time spent in each male compartment; and 5) which males she mated with during the trial. Timing of copulation and intromission were not analysed as mating pairs often moved in and out of nest boxes during copulation. A visit involved the female stopping to look, sniff, chew or climb on male doors and doorsteps and did not include the female walking past doors without stopping. Female visits that lasted five seconds or longer were timed. Behaviours that included male/female and female/female agonistic encounters, scent marking, chasing and sexual positions [36,37] were counted as distinct bouts.

### Genetic analyses

Prior to each experiment, animals were genotyped using seven microsatellite markers as described in Parrott et al. [30,31]. Relatedness between all members of the captive colony was determined using the GENEPOP 3.4 program to analyse allele frequencies and Kinship 1.3.1 to give a numerical score. Kinship values in relation to each female were used when choosing females and their four potential mates in this experiment. Mean (± SE) Kinship values were 0.14 ± 0.02 (median 0.12, range -0.07–0.38) for the two more genetically similar and -0.10 ± 0.01 (median -0.10, -0.31–0.09.) for the two more genetically dissimilar males compared to each female over both years and this difference was significant for each female (paired t-test t = -16.87, p <0.001). Female pairs in each experiment differed in genetic relatedness to each other and males differed in relatedness to each of the females. This allowed each female different choices of mates that were genetically dissimilar or similar to themselves.

Pouch young born from matings during these experiments were genotyped at five microsatellite loci using DNA extracted from tail tip samples (<1 mm of skin) taken at four weeks of age [30,31]. The paternity of each pouch young was allocated using the CERVUS 2.0 program with 100% confidence.

### Analysis of results

Males were divided into either the genetically similar (2 males/female) or genetically dissimilar (2 males/female) categories based on Kinship values described above for analyses of female choice and paternity. Efforts were made to reduce pseudoreplication in the dataset, though this was not always possible. Comparisons between the measures of female behaviour directed toward similar verses dissimilar males and the reproductive outcomes were performed using either repeated measures ANOVA to correct for between-individual differences or chi-square tests (when the dependent variable was binary) using the statistical program SYSTAT [38]. Weights of individuals that produced offspring and those that did not were compared using t-tests.

## Results

### Mate choice

#### Investigation by females

**Fig 2:**
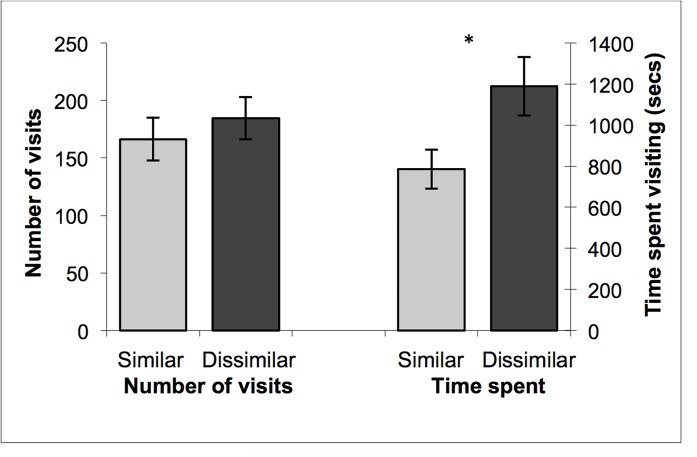
The number of visits and time spent at male doors. The mean (± SE) number of times female agile antechinus (n = 28) visited the doors of males that were more genetically similar and more dissimilar to themselves (left) and the mean (± SE) time (seconds) female agile antechinus (n = 28) spent visiting the doors of males that were more genetically similar and more dissimilar to themselves (right). An asterisk (*) indicates a significant difference from the other value (p = 0.003).

All but one female (27/28) visited the four male doors prior to focussing on a preferred male(s). There was no significant difference in the number of times a female visited the door of the males that were more genetically similar or dissimilar to herself (F_1,26_ = 2.46, p = 0.13; Fig 2). However, females spent significantly more time investigating the doors of males that were genetically dissimilar to themselves (F_1,26_ = 11.05, p = 0.003; Fig 2). Once interested in a particular male(s), females would chew, push and climb on doors of these males prior to gaining access. Genetically dissimilar males attracted significantly more bouts of chewing, pushing and climbing behaviours than similar males (mean ± SE per female, Similar: 9.1 ± 1.7 times; Dissimilar: 16.2 ± 3.4 times; F_1,26_ = 6.50, p = 0.017).

Females investigated males that were acting in an aggressive or vocal manner from a distance, returning to examine them after being chased from and/or grabbed through doors. There was no difference in the number of chases/attacks from genetically similar or dissimilar males (mean ± SE per female, Similar: 9.8 ± 1.4; Dissimilar: 11.8 ± 2.0; F_1,26_ = 0.75, p = 0.39). Most females that were seized by males through doors were able to quickly free themselves (67%, n = 30 times), while others were released after observer intervention (33%, n = 15 times). No females attempted to enter compartments with males vocalising or acting in an aggressive manner (n = 0/28 females).

#### Entries to male compartments

**Fig 3:**
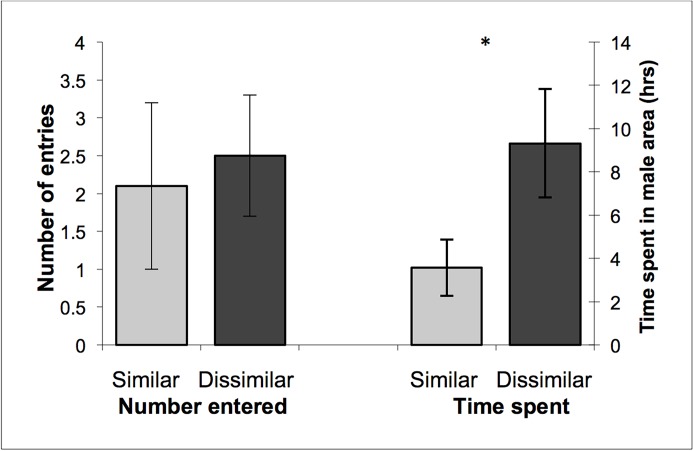
The number of entries and time spent in male enclosures. The mean (± SE) number of times female agile antechinus (n = 28) entered into the compartments of males that were more genetically similar and more dissimilar to themselves (left) and the mean (± SE) time (hours) female agile antechinus (n = 21) spent in the compartments of males that were more genetically similar and more dissimilar to themselves (right). An asterisk (*) indicates a significant difference from the other value (p = 0.046).

Females entered into the compartments of both genetically similar and dissimilar males and there was no difference in the number of times they did so (Repeated measures ANOVA; F_1,26_ = 0.29, p = 0.60; Fig 3). However, females typically spent more than double the time in the enclosures of genetically dissimilar males (F_1,26_ = 4.38, p = 0.046; Fig 3). Half the females (14/28) entered male compartments more than once with two females entering different male compartments a combined total of 41 and 32 times respectively (mean ± SD = 4.64 ± 9.45; Table 1).

**Table 1:** Overview of female visits, entries, matings and pouch young produced.

	Number of females	Additional data
Entry into 1 male compartment	14/28	
Entry into >1 male compartment	14/28	
Actively seeking mate and entered male nest box	21/28	7 females entered the male area, but fled from the male when approached. 2 females were rejected by males despite attempts to gain male attention.
Mated with 1 male	13/28	6/13 females produced young
Mated with >1 male	6/28	5/6 females produced young
Failed to mate	9/28	
Produced pouch young	11/28	Total of 47 young produced (range 1–9 PY/litter; mean ± SE litter size 4.27 ± 0.79)

The number of females that entered into one, or more than one, male compartment, sought to mate with males, mated with single or multiple males and produced pouch young, including additional data on female behaviour and the number of young produced.

### Genetic relatedness and mating behaviour

**Fig 4:**
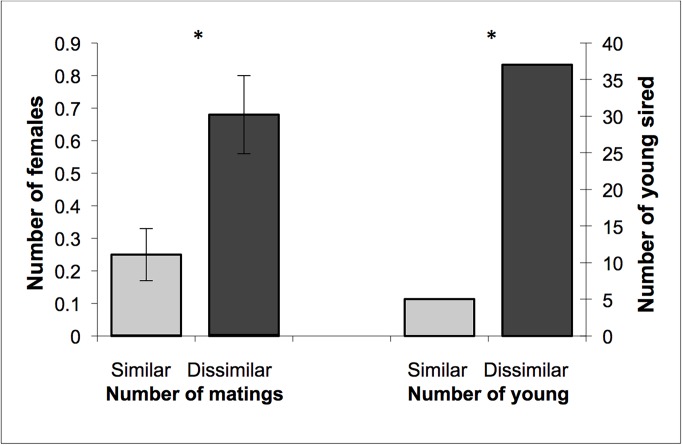
The number females that mated with genetically similar and dissimilar males and paternity of young produced. The mean (± SE) number of females that mated with the more genetically similar and more dissimilar males (left), and the number of agile antechinus young sired by the more genetically similar and more dissimilar males. Asterisks (*) indicate significant differences in pairs of values (number of matings, p <0.001; number of young, p < 0.016).

Females actively sought males and entered into nest-boxes with males of their own accord (n = 21). Females often mated with a male multiple times before leaving his compartment (n = 11 females), but it was not possible to score the exact number of matings during each visit. Some females (n = 6) chose to enter and mate with more than one male, but most females mated with only one male (n = 13) and 9 females failed to mate (Table 1). Four females re-entered male compartments and mated with the same male up to 5 times. Some of these re-entries (n = 3 females) were sequential, while one was after mating with different males. Females were more likely to mate with one or both of the more genetically dissimilar males (17/28) than with one or both of the more genetically similar males (7/28; X^2^ = 7.29, df = 1, p = 0.007; Fig 4). Females that mated with more than one male did not appear to trade up to more genetically dissimilar males with four females mating with the more genetically dissimilar male first, one mating with the more similar of their two males first, and one female mating with a similar male in between two more genetically dissimilar males. Some males in each year (2003: n = 2/12; 2004: n = 2/12) were disproportionately popular, regardless of genetic relatedness and were chosen by all females they encountered.

Females did not appear to follow each other and entered into the same male compartment simultaneously in only three trials. In two of those trials females pushed, chased and bit each other until one left from the males’ nest-boxes and compartments. Both females that were chased from a male compartment later re-entered the compartment and one stayed to mate with the male. Female agonistic behaviour was observed only near males with low levels occurring during or following mating events, except in one instance where it also occurred near the female nest-tube and food trays. Females chose to mate with the same male in one trial only, with one of the females in that trial mating with 3 of the four males available.

#### Male behavior

All males (n = 24) scent marked their compartments using urine and paracloacal and cutaneous sternal glands. Scent marking behaviour and wet scent-marked areas were most often apparent near the door areas where females had scent-marked and on the upright climbing lattices. Males appeared to show interest in and accept most females regardless of whether the female showed passive or agonistic (hissing and biting) behaviours, but ignored the advances of others. Females were able to enter the compartments and nest-boxes of these males while the male was awake without any male reaction (n = 6 females). Three of these females pushed and climbed over males and assumed mating positions, but did not elicit a response and left soon after. Four females that were rejected by some males were accepted by others. Two females were rejected by all males, but the males in these trials mated with the other female present, showing that these males were interested in females and capable of mating. The two females ignored by all males were within their most fertile receptive period and were within the weight range of females mated by males, though were two of the lighter females that year (rejected females: 14.4 and 14.8 g; mean of all females in 2003 = 15.1 ± 0.22, range = 14–17 g).

### Offspring production and genetic relatedness

In 2003, 6 females gave birth to 28 young following this experiment. Samples were taken from 23 pouch young (5 young were lost before they were large enough to sample). In 2004, 5 females gave birth to 19 young following these experiments, all of which were sampled (Table 1). Females that produced litters were mated in their most fertile period (n = 8) or towards the end their receptive period (n = 3). Females that did not give birth were either in (n = 14), or at the beginning of their most fertile period (days 4–6; n = 3), and nine of those females failed to mate. There was no difference in weight between females that produced young (16.4 ± 0.5 g) and did not produce young (15.6 ± 0.4 g; t = 1.30, p = 0.21), or in males that sired (26.2 ± 0.6 g) or did not sire young (27.4 ± 0.8 g; t = -1.19, p = 0.25).

Of the 19 females that were observed to have mated, offspring were produced by 5 of the 6 that had mated with more than one male and 6 of the 13 that had mated with only one male (X^2^ = 2.33, df = 1, p = 0.13). Of the 11 females that produced young, mean litter size was 4.66 ± 1.05 among females that mated to one male and 2.80 ± 0.73 among females that mated to more than one male (ANOVA; F_1,9_ = 1.94, p = 0.20). Of the 13 females that mated with only one male, offspring were produced by 6/11 that mated with a more dissimilar male and 0/2 that mated with a more similar male (X^2^ = 2.03, df = 1, p = 0.16). Multiple paternity was observed in 2/11 litters with two fathers in each. Of the four females that mated with both dissimilar and similar males and produced offspring, the dissimilar mates sired more young on average in two cases and the similar mate more young in the other two cases.

Overall, females were more likely to produce offspring with genetically dissimilar males (10/28) than similar males (2/28; X^2^ = 6.79, df = 1, p = 0.01) and produced, on average, more young with their pair of dissimilar males (1.32 ± 0.44) than similar males (0.19 ± 0.13; F_1,54_ = 6.24, p = 0.016). In total, 88% of young produced were sired by the genetically dissimilar males. Two males sired young in two litters, while nine males sired a litter with one female.

## Discussion

This study has shown for the first time that female agile antechinus actively seek, and are receptive to, matings with more than one male and that mate choice is an important strategy in the antechinus breeding system. Although females watched and interacted with all the males, they spent significantly more time investigating males that were genetically dissimilar to themselves. Females also mated and produced young with genetically dissimilar males significantly more times than with the genetically similar males.

Female agile antechinus in wild populations almost always produce litters that are sired by more than one male ([14], MLP unpub. data). Here, despite the availability of four males, the majority of females chose to be monandrous, often returning to mate with a single male multiple times. This differs to the field data and may suggest that when constraints exerted by males are relieved, females avoid multiple matings. Females that mate with more than one male may hedge their bets against the possibility of a mate being sterile or incompatible [2,39], but females in this study chose males that were more genetically dissimilar to themselves and so avoided mating with males more likely to be genetically incompatible. In this study, genetic relatedness was determined using seven microsatellite markers, thus questions remain regarding the levels of relatedness between potential mates. Further research with additional markers and research into genes of the Major Histocompatibility Complex could be used to further clarify relationships in the future. Females may also mate with an available male and then ‘trade up’ by mating with a higher quality male if encountered [40,41]. Each trial in this experiment was run for 72 hours, so offered the opportunity for females to trade up to higher quality available males. However, the majority of females in this experiment that mated with more than one male did so with a more dissimilar male first and showed the highest level of interest in their first mate. As all males were simultaneously available, this may not be indicative of a wild situation where females may be expected to encounter new males at different times during their long receptive period. Last male sperm precedence operates in the agile antechinus [26,32], so females may be able to influence the paternity of their young by trading up to genetically superior males during their most fertile periods [42]. The small litter sizes produced in this study may have resulted in the decreased incidence of mixed paternity when compared with wild data. However, all but one female that mated with more than one male produced young, while less than half the females that mated with one male produced a litter, suggesting that females that mate with multiple partners increase their reproductive success. Research has shown that female brown antechinus (*Antechinus stuartii*) that mate with multiple males during a single receptive period produce significantly more young than females allowed to mate with only one male [43]. A similar effect has been observed in European adders (*Vipera berus*), where females that mated with more than one male had fewer stillborn young [44]. In sand lizards, increased number of mates correlated with increased egg-hatching success and survival of young [45], while, female blue tits (*Parus caeruleus*) and tree swallows (*Tachycineta bicolor*) increase the heterozygosity and thus the potential fitness and reproductive success of their offspring through additional extra-pair matings [46,47]. Conversely, females may avoid mating with multiple males to reduce the risk of parasite transmission, illness or injury sustained during mating [20]. Here, females avoided males that were particularly vocal or aggressive at their doors, regardless of the level of genetic dissimilarity between the pair. The relationships between female mate choice, male coercion and reproductive success are complex and warrant further investigation.

Males that were genetically dissimilar to females obtained more matings than genetically similar males and sired more young, as has been observed in a variety of taxa [6,1,10]. However, compared with the number of matings obtained by males in each category, genetically dissimilar males sired a disproportionately higher number of young than genetically similar males per mating event. Previous research by Kraaijeveld-Smit et al. [32] suggested that spermatozoa from genetically dissimilar males may be more successful due to sperm competition [40]. Female agile antechinus store sperm in specialised isthmic crypts in their oviducts for up to 15 days [13,34,48] providing time and a suitable environment for sperm competition. Potentially, males that are genetically dissimilar to females are not only chosen pre-copulation, but their spermatozoa also compete more successfully post-copulation by cryptic female selection of sperm within the reproductive tract [40,49,50,51]. It is possible that part of the uterine mortality encountered in this species which progressively reduces viable embryos to 60% by the neurula stage [34] is due to matings between genetically similar individuals. In natural populations, larger males may also secure more matings and sire more young ([14], MLP unpub data), but *ex situ* research into female mate choice shows that female agile antechinus do not choose males based on size [30]. Regardless, the effect of male size on mate selection in this experiment was excluded as a confounding factor by selection of males of similar sizes. There was no evidence of mate copying, as occurs in species including the guppy [52] and sage grouse (*Centrocercus urophasianus*; [53]) where females copy the preferences of other females, even changing from their original choice [52]. Although female antechinus entered the enclosures of the same males, two females chose to mate with the same male in only one of 14 trials. One male sired young in two litters, but all other sires produced one litter each. Due to the 72 hour time period of the trials, females had time to access all males, regardless of whether another female had chosen the male.

Female antechinus can determine the difference between scents from more and less genetically similar males and prefer chemosensory cues from genetically dissimilar males [31], suggesting that the process of mate choice in this experiment was influenced by these cues (see review in [54]). Although important, genetic relatedness between mates may be only one aspect of a set of mate preference criteria used by females, particularly in the wild. Some males in this experiment were preferred by all females they encountered, regardless of the level of genetic relatedness. This occurred in both years, suggesting that it was not an anomaly and that certain traits possessed by some males that we were not able to identify in this study may override the importance of genetic relatedness.

Following this experiment, 47 young were born to 11 mothers. This was fewer than expected and differs from wild populations in which all teats are generally occupied [55,56]. There are two likely reasons for this outcome. Firstly, animals used in this experiment were collected during severe drought conditions which significantly decreased weight, survival and litter sizes in the wild [33]. This probably also influenced fertility in the captive population used in this study, despite the availability of increased nutrition, because animals were collected less than one month prior to the breeding season and were in poor condition [33]. Secondly, most litters (8) were produced from matings in the most fertile period of receptivity, with the remaining three produced from matings late in the receptive period. No young were produced from females paired on days 4–6 of their receptive period. This concurs with the findings of Selwood and McCallum [13] who showed that matings that occurred more than 14 days, or less than 5 days, from the spontaneous ovulation resulted in low numbers of normal fertile embryos and few young. In antechinus and some other dasyurid marsupials oestrus is difficult to define [35]. Females may be receptive to mating at times when conception is unlikely (eg too early or late in respect to ovulation, or even during gestation) and the female may not be fertile [35]. Selwood and McCallum [13] demonstrated that for single inseminations, sperm survival time is finite. For single inseminations outside that period ie 0 to 4 days before ovulation and 14–20 days before ovulation, the percentage of normal embryos is 0 to 58% and the averages for these periods are 44.5 and 27% respectively [13]. Thus, some females in this study mated outside their period of optimum fertility which is likely to have influenced their reproductive successs. Additionally, previous studies have shown that antechinus can have a lower breeding success in captivity than in the wild (e.g. [57]).

Male mate choice has received less attention than mate choice by females, but may also be important [58]. Mate choice by males may occur when there is a female-bias in the operational sex ratio [59], when females show secondary sexual characteristics such as colour or ornamentation as seen in a variety of birds and fish [60,61,62], when there is a preference for novel over resident females [63], when female fertility is correlated with her body size [64] and/or choice may be based on genetic relatedness [65]. Here, we describe the first case of male mate choice in a marsupial to our knowledge, with male antechinus appearing disinterested in some females and ignoring their efforts to gain attention. Males prefer novel females rather than familiar previously-mated females in green anole lizards (*Anolis carolinensis*; [64]), but familiarity with the female did not appear to influence male mate choice in the agile antechinus. Males re-mated with the same females if they stayed with them or re-entered the compartment. This was unexpected as males have a relatively small and finite number of spermatozoa available for insemination [66] and may be expected to maximise the number of females inseminated to increase their siring success. Male mate choice also did not appear to be affected by his level of genetic relatedness to the female nor by her fertility status which can be an influence in some species [67]. In oldfield mice (*Peromyscus polionotus rhoads*), males paired with preferred females had a greater siring success than those paired with non-preferred females based on compatibility of mates [68]. Here, females that were rejected by some males were accepted by others and successfully produced young, suggesting compatibility, rather than the fertility or attractiveness of the female, affected male choice. Female agonistic behaviour did not appear to deter males, a similar observation to that made by Shimmin et al. [37], and female body mass also did not appear to influence male choice or female reproductive success in this experiment with the lightest and heaviest females mating and no differences in weight between females that did and did not produce young. The reason(s) for the preference by male agile antechinus of certain females over others is not clear. The role of male mate choice and its effects on breeding success in the agile antechinus and other species warrants further examination.

This research has provided new and important insights into the effects of genetic relatedness and female mate choice on siring success. It also provides new knowledge about the unusual mating system of the agile antechinus. Future studies of mate choice and its effects on reproductive success will shed light on the evolution of the mating system of the agile antechinus, which provides an interesting and useful paradigm for studies in other related species.

## References

[1] Neff BD, Pitcher TE (2005) Genetic quality and sexual selection: an integrated framework for good genes and compatible genes. Molecular Ecology 14: 19–38. 15643948 10.1111/j.1365-294X.2004.02395.x

[2] Tregenza T, Wedell N (2000) Genetic compatibility, mate choice and patterns of parentage: invited review. Molecular Ecology 9: 1013–1027. 10964221 10.1046/j.1365-294x.2000.00964.x

[3] Drickamer LC, Gowaty PA, Holmes CM (2000) Free female mate choice in house mice affects reproductive success and offspring viability and performance. Animal Behaviour 59: 371–378. 10675259 10.1006/anbe.1999.1316

[4] Jones TM, Quinnell RJ, Balmford A (1998) Fisherian flies: benefits of female choice in a lekking sandfly Proceedings of the Royal Society of London, Series B 265 1651–1657.

[5] Consuegra S, Garcia de Leaniz C (2008) MHC-mediated mate choice increases parasiteresistance in salmon. Proceeding of the Royal Society of London B. 275: 1397–1403. 10.1098/rspb.2008.0066 18364312 PMC2602703

[6] Mays HL, Hill GE (2004) Choosing mates: good genes versus genes that are a good fit. Trends in Ecology and Evolution 19: 554–559. 16701321 10.1016/j.tree.2004.07.018

[7] Puurtinen M, Ketola T, Kotiaho JS (2009) The good-genes and compatible-genes benefits of mate choice. The American Naturalist 174: 741–752. 10.1086/606024 19772439

[8] Charpentier MJE, Crawford JC, Boulet MN, Drea CM (2010) Message 'scent': lemurs detect the genetic relatedness and quality of conspecifics via olfactory cues. Animal Behaviour 80: 101–108.

[9] Kempanears B (2007) Mate choice and genetic quality: A review of the heterozygosity theory. Advances in the Study of Behavior 37: 189–278.

[10] Mays HL, Albrecht T, Liu M, Hill GE (2008) Female choice for genetic complementarity in birds: a review. Genetica. 34 147–158. 10.1007/s10709-007-9219-5 17973192

[11] Stockley P, Searle JB, Macdonald DW, Jones CS (1993) Female multiple mating behaviour in the common shrew as a strategy to reduce inbreeding. Proceedings of the Royal Society of London, Series B 254: 173–179. 8108451 10.1098/rspb.1993.0143

[12] Hoogland JL (1998) Why do female Gunnison’s prairie dogs copulate with more than one male? Animal Behaviour 55: 351–359. 9480703 10.1006/anbe.1997.0575

[13] Selwood L, McCallum F (1987) Relationship between longevity of spermatozoa after insemination and the percentage of normal embryos in brown marsupial mice (Antechinus stuartii). Journal of Reproduction and Fertility 79: 495–503. 3572881 10.1530/jrf.0.0790495

[14] Kraaijeveld-Smit FJL, Ward SJ, Temple-Smith PD (2002a) Multiple paternity in a field population of a small carnivorous marsupial, the agile antechinus, Antechinus agilis. Behavioural Ecology and Sociobiology 52: 84–91.

[15] Parrott ML, Ward SJ, Taggart DA (2005) Multiple paternity and communal maternal care in the feathertail glider (Acrobates pygmaeus). Australian Journal of Zoology 53: 79–85.

[16] Hill CE, Gjerdum C, Elphick CS (2010) Extreme Levels of Multiple Mating Characterize the Mating System of the Saltmarsh Sparrow (Ammodramus Caudacutus). The Auk 127: 300–307.

[17] Meister B, Ursenbacher S, Baur B (2012) Frequency of multiple paternity in the grass snake (Natrix natrix) Amphibia-Reptilia. 33: 308–312.

[18] Noble DWA, Keogh JS, Whiting MJ (2013) Multiple mating in a lizard increases fecundity but provides no evidence for genetic benefits. Behavioural Ecology 24: 1128–1137.

[19] Arnqvist G (1988) On multiple matings and female fitness: comments on Loman et al.. Oikos 54: 248–250.

[20] Stockley P (1997) Sexual conflict resulting from adaptation to sperm competition. Trends in Ecology and Evolution 12: 154–159. 21238013 10.1016/s0169-5347(97)01000-8

[21] Brown JL (1997) A theory of mate choice based on heterozygosity. Behavioural Ecology 8: 60–65.

[22] Jennions MD (1997) Female promiscuity and genetic incompatibility. Trends in Ecology and Evolution 12: 251–253. 21238060 10.1016/s0169-5347(97)01128-2

[23] Loman J, Masden T, Hakansson T (1988) Increased fitness from multiple matings, and genetic heterogeneity: a model of a possible mechanism. Oikos 52: 69–72.

[24] Parker GA (1970) Sperm competition and its evolutionary consequences in the insects. Biological Reviews 45: 526–567.

[25] Slatyer RA, Mautz BS, Backwell PRY, Jennions MD (2012) Estimating genetic benefits of polyandry from experimental studies: a meta-analysis. Biological Reviews 87: 1–33. 10.1111/j.1469-185X.2011.00182.x 21545390

[26] Shimmin GA, Taggart DA, Temple-Smith PD (2000) Sperm competition and genetic diversity in the agile antechinus (Dasyuridae: Antechinus agilis). Journal of Zoology 252: 343–350.

[27] Scott MP, Tan TN (1985) A radiotracer technique for the determination of male mating success in natural populations. Behavioural Ecology and Sociobiology 17: 29–33.

[28] Lazenby-Cohen KA, Cockburn A (1988) Lek promiscuity in a semelparous mammal, Antechinus stuartii (Marsupialia, Dasyuridae). Behavioural Ecology and Sociobiology 22: 195–202.

[29] Kraaijeveld-Smit FJL, Ward SJ, Temple-Smith PD (2003) Paternity success and the direction of sexual selection in a field population of a semelparous marsupial, Antechinus agilis. Molecular Ecology 12: 475–484. 12535097 10.1046/j.1365-294x.2003.01745.x

[30] Parrott ML, Ward SJ, Temple-Smith PD (2006) Genetic similarity, not male size, influences female mate choice in the agile antechinus (Antechinus agilis). Australian Journal of Zoology 54: 319–323.

[31] Parrott ML, Ward SJ, Temple-Smith PD (2007a) Olfactory cues, genetic relatedness and female mate choice in the agile antechinus (Antechinus agilis). Behavioural Ecology and Sociobiology 61: 1075–1079.

[32] Kraaijeveld-Smit FJL, Ward SJ, Temple-Smith PD, Paetkau D (2002b) Factors influencing paternity success in Antechinus agilis: last-male sperm precedence, timing of mating and genetic compatibility. Journal of Evolutionary Biology 15: 100–107.

[33] Parrott ML, Ward SJ, Temple-Smith PD, Selwood L (2007b) Effects of drought on weight, survival and breeding success of agile antechinus (Antechinus agilis), dusky antechinus (A. swainsonii) and bush rats (Rattus fuscipes). Wildlife Research 34: 437–442.

[34] Selwood L (1982) Brown antechinus, Antechinus stuartii: management of breeding colonies to obtain embryonic material and pouch young In: Evans DD, editor. The management of Australian mammals in captivity. Australia: Zoological Board of Victoria pp. 31–36.

[35] Selwood L (1985) Synchronisation of oestrus, ovulation and birth in female Antechinus stuartii (Marsupialia: Dasuridae). Australian Mammalogy 8: 91–96.

[36] Marlow BJ (1961) Reproductive behaviour of the marsupial mouse, Antechinus flavipes (Waterhouse) (Marsupialia) and the development of the pouch young. Australian Journal of Zoology 9: 203–220.

[37] Shimmin GA, Taggart DA, Temple-Smith PD (2002) Mating behaviour in the agile antechinus Antechinus agilis (Marsupialia: Dasyuridae). Journal of Zoology 258: 39–48.

[38] SYSTAT. (2004) SYSTAT for Windows, statistics, version 11 Illanois: SYSTAT.

[39] Keller L, Reeve HK (1995) Why do females mate with multiple males? The sexually selected sperm hypothesis. Advances in the Study of Behaviour 24: 291–315.

[40] Jennions MD, Petrie M (2000) Why do females mate multiply? A review of the genetic benefits. Biological Reviews 75: 21–64. 10740892 10.1017/s0006323199005423

[41] Pitcher TE, Neff BD, Rodd FH, Rowe L (2003) Multiple mating and sequential mate choice in guppies: females trade up. Proceedings of the Royal Society of London B. 270: 1623–1629. 12908984 10.1098/rspb.2002.2280 PMC1691420

[42] Birkhead T, Møller A (1993) Female control of paternity. Trends in Ecology and Evolution 8: 100–104. 10.1016/0169-5347(93)90060-3 21236119

[43] Fisher DO, Double MC, Blomberg MD, Cockburn A (2006) Post-mating sexual selection increases lifetime fitness of polyandrous females in the wild. Nature 444: 89–92. 17080089 10.1038/nature05206

[44] Madsen T, Shine R, Loman J, Hakansson T (1992) Why do female adders copulate so frequently? Nature 355: 440–441.

[45] Olsson M, Gullberg A, Tegelström H, Madsen T, Shine R (1994) Can female adders multiply? Nature 369: 528.

[46] Foerster K, Delhey K, Johnsen A, Lifjeld JT, Kempenaers B (2003) Females increase offspring heterozygosity and fitness through extra-pair matings. Nature 425: 714–717. 14562103 10.1038/nature01969

[47] Stapleton MK, Oddmund K, Lifjeld JT, Robertson RJ (2007) Female tree swallows (Tachycineta bicolor) increase offspring heterozygosity through extrapair mating. Behavioral Ecology and Sociobiology. 61: 1725–1733.

[48] Shimmin GA, Jones M, Taggart DA, Temple-Smith PD (1999) Sperm transport and storage in the agile antechinus (Antechinus agilis). Biology of Reproduction 60: 1353–1359. 10330092 10.1095/biolreprod60.6.1353

[49] Zeh JA, Zeh DW (1996) The evolution of polyandry I: intragenomic conflict and genetic incompatibility. Proceedings of the Royal Society of London, Series B 263: 1711–1717.

[50] Zeh JA, Zeh DW (1997) The evolution of polyandry II: post-copulatory defenses against genetic incompatibility. Proceedings of the Royal Society of London, Series B 264: 69–75.

[51] Nordeide JT (2007) Is there more in ‘gamete quality’ than quality of the gametes? A review of effects of female mate choice and genetic compatibility on offspring quality. Aquaculture Research 38: 1–16.

[52] Dugatkin LA, Godin JJ (1992) Reversal of Female Mate Choice by Copying in the Guppy (Poecilia reticulata) Proceedings of the Royal Society of London B. 249: 179–184. 10.1098/rspb.1992.0101 1360679

[53] Gibson RM, Bradbury JW, Sandra L, Vehrencamp SL (1991) Mate choice in lekking sage grouse revisited: the roles of vocal display, female site fidelity, and copying. Behavioral Ecology. 2: 165–180.

[54] Johansson BG, Jones TM (2007) The role of chemical signals in sexual selection. Biological Reviews of the Cambridge Philosophical Society 82: 265–289. 17437561 10.1111/j.1469-185X.2007.00009.x

[55] Woolley PA (1966) Reproduction in Antechinus spp., and other dasyurid marsupials. Symposia of the Zoological Society of London 15: 281–294.

[56] Wood DH (1970) An ecological study of Antechinus stuartii (Marsupialia) in a south-east Queensland rainforest. Australian Journal of Zoology 18: 185–207.

[57] Aslin HJ (1982) Small dasyurid marsupials: their maintenance and breeding in captivity In: Evans DD, editor. The management of Australian mammals in captivity. Australia: Zoological Board of Victoria pp. 22–26.

[58] Edward DA, Chapman T (2011) Review: The evolution and significance of male mate choice. Trends in Ecology & Evolution 26: 647–654. 21890230 10.1016/j.tree.2011.07.012

[59] Kvarnemo C, Ahnesjo I (1996) The dynamics of operational sex ratios and competition for mates. Trends in Ecology and Evolution 11: 404–408. 21237898 10.1016/0169-5347(96)10056-2

[60] Hill GE (1993) Male mate choice and the evolution of female plumage colouration in the house finch. Evolution. 47: 1515–1525. 28564892 10.1111/j.1558-5646.1993.tb02172.x

[61] Nolan PM, Dobson FS, Nicolaus M, Karels TJ, McGraw KJ, Jouventin P (2010) Mutual Mate Choice for Colorful Traits in King Penguins. Ethology. 116: 635–644.

[62] Amundsen T, Forsgren E (2001) Male mate choice selects for female coloration in a fish. Evolution 98: 13155–13160. 10.1073/pnas.211439298 PMC60840 11606720

[63] Orrell KS, Jenssen TA (2002) Male mate choice by the lizard Anolis carolinensis: a preference for novel females. Animal Behaviour. 63: 1091–1102

[64] Orrell KS, Jenssen TA (2002) Male mate choice by the lizard Anolis carolinensis: a preference for novel females. Animal Behaviour: 1091–1102.

[65] Ryan KK, Lacy RC (2003) Monogamous male mice bias behaviour towards females according to very small differences in kinship. Animal Behaviour. 65: 379–384.

[66] Taggart DA, Temple-Smith PD (1990) Effects of breeding system and mating on total number and distribution of spermatozoa in the epididymis of the brown marsupial mouse, Antechinus stuartii. Journal of Reproduction and Fertility 88: 81–91. 2313656 10.1530/jrf.0.0880081

[67] Craig AS, Herman LM, Pack AA (2002) Male mate choice and male-male competition coexist in the humpback whale (Megaptera novaeangliae). Journal of Zoology 80: 745–755.

[68] Ryan KK, Altmann J (2001) Selection for male choice based primarily on mate compatibility in the oldfield mouse, Peromyscus polionotus rhoadsi. Behavoural Ecology and Sociobiology. 50: 436–440.

